# Cluster analysis with body composition data for health risk assessment in children

**DOI:** 10.1038/s41390-025-04447-6

**Published:** 2025-10-30

**Authors:** Wataru Kudo, Keita Terui, Midori Yamamoto, Rieko Takatani, Aya Hisada, Chisato Mori, Tomoro Hishiki, Kenichi Sakurai

**Affiliations:** 1 Department of Pediatric Surgery, Graduate School of Medicine, Chiba University, Chiba, Japan; 2 Division of Pediatric Surgery, Department of Surgery, Jichi Medical University, Shimotsuke, Japan; 3 Department of Sustainable Health Science, Center for Preventive Medical Sciences, Chiba University, Chiba, Japan; 4 Faculty of Education, Chiba University, Chiba, Japan; 5 Department of Bioenvironmental Medicine, Graduate School of Medicine, Chiba University, Chiba, Japan; 6 Department of Nutrition and Metabolic Medicine, Center for Preventive Medical Sciences, Chiba University, Chiba, Japan

## Abstract

**Background:**

This study investigated populations of children at increased health risks using an integrated analysis of anthropometric/body composition data.

**Methods:**

A cross-sectional study of elementary school students (first–sixth grade) was conducted from 2020 onward. Body composition measurements using bioelectrical impedance method, anthropometric measurement, and sub-measures (abdominal circumference, serum lipid levels, activity level, and sleep duration) were performed. Measurements were repeated at 1 and 2 years. Body composition data were standardized using polynomial regression models, and hierarchical clustering analysis was performed to identify subpopulations.

**Results:**

Reference value models were constructed using 917 standardized body composition data. Cluster analysis of standardized body composition data with body mass index (BMI) identified five clusters. Two high BMI clusters were identified: one characterized by high fat and muscle mass, and the other by high fat but average muscle mass. Cluster classification revealed significant effects for body fat percentage, abdominal circumference, lipid-related indicators, and sleep duration. The subpopulation with high fat/average muscle mass had high body fat percentage, large abdominal circumference, high lipid-related indices, and short sleep duration.

**Conclusions:**

Anthropometric and body composition data integration identified a subgroup of children at increased health risk, highlighting the importance of incorporating body composition assessment into routine childhood physical examinations.

**Impact:**

- We established reference data for fat mass, lean body mass, muscle mass, bone mass, and total water mass indices in children aged 7 to 14 years.
- Integrating anthropometric and body composition data allowed effective identification of pediatric subpopulations at increased health risks, even in generally healthy populations.
- Children with a high fat mass but average muscle mass had a high body fat percentage, large abdominal circumference, poor lipid profiles, and shorter sleep duration.
- Incorporating body composition analysis into standard health checkups for children could facilitate the identification of groups at high risk of health problems.

## Introduction

Body weight is commonly used as an indicator of health. Indeed, being underweight, overweight, or obese during childhood and adolescence is considered to exert a negative impact on health throughout life.^1,2^ Among the different body weight components, muscle mass accounts for the largest proportion of mass in children.^3–5^ Problems arising from the loss of muscle mass are the same as those arising from sarcopenia, and are not fully related to body weight. In the adult or older population, sarcopenia is not limited to individuals classified as underweight,^6–8^ but also occurs in those classified as obese (sarcopenic obesity), and has a negative impact on health.^9–12^ This phenomenon results in an imbalance in body composition (BC), suggesting that body weight and muscle mass alone are not the best health indicators. Thus, to accurately assess health risks, it is important to evaluate BC, including muscle and fat mass, in addition to body weight.

In children, the muscle-to-fat ratio (MFR) is a measure of BC imbalance associated with sarcopenic obesity,^13^ metabolic syndrome,^14^ and nonalcoholic fatty liver disease,^15^ and is used as a marker of health-related fitness.^16^ However, because of the negative correlation between MFR and body mass index (BMI),^14,15^ further stratification within high and low BMI groups is challenging. Torres-Costoso et al.^17,18^ previously stratified MFR in addition to lean body mass index (LMI) in children, performing machine learning cluster analysis using LMI and fat BMI to identify five clusters in young adults. Identifying pediatric populations at high risk of health complications would enable the provision of personalized interventions; however, methods to better identify such pediatric populations remain unestablished.

In the present study, we hypothesized that a comprehensive analysis of BC would allow more appropriate identification of subpopulations of children. To investigate this, we comprehensively analyze BC using a bioelectrical impedance method in generally healthy children to define different subpopulations. Using machine-learning techniques, we propose a reference value model for BC; introduce a novel classification method; and explore the associations between BC, body shape, activity level, and biochemical markers in children.

## Methods

### Study design and participants

This single-center cross-sectional study of school children was performed at an elementary school affiliated with a university in Japan. The study protocol is available on our institution’s website (access link: https://cpms.chiba-u.jp/en/research/Study%20protocol.pdf). In December of the financial year (FY) 2020, children in grades 1–6 were invited to participate in the study, resulting in the enrollment of 355 children. Although there were no specific exclusion criteria in the study protocol, children aged 6 years were excluded because of their small sample size (*n* = 7). Participants underwent physical measurements; BC analysis using bioelectrical impedance analysis (BIA); abdominal circumference (AC) measurement; blood tests; and monitoring of steps, activity, and sleep duration using a wrist-worn device from January to February 2021 for FY2020. These measurements were further conducted for FY2021 (December 2021 to March 2022) and FY2022 (December 2022 to February 2023). Among the 355 enrolled children, 2 children were excluded due to incomplete BC data, resulting in a final analytical cohort of 353 children who underwent at least one BC measurement during the 3-year study period.

The study protocol was reviewed and approved by the Ethics Committee of the Graduate School of Medicine, Chiba University (3909). Written informed assent and consent were obtained from all children who participated in the study and their parents. This study was conducted in accordance with the ethical guidelines for medical research in Japan and the principles of the Declaration of Helsinki. This study was registered as an observational study in the UMIN Clinical Trials Registry (registration no. UMIN000046126) and followed the Strengthening the Reporting of Observational Studies in Epidemiology reporting guidelines.^19^

### Measurements and data manipulation

Data pertaining to anthropometry, BC, AC, lipid-related indicators, number of steps, and activity and sleep durations were collected for each participant.

### Anthropometric and BC data

Height was measured in the barefoot standing position and visually recorded to one decimal place (height in cm). BC analysis was performed using MC-780A (TANITA, Tokyo, Japan). At the time of BC analysis, participants wore school gymnastic clothes, with the weight of clothes standardized at 0.5 kg for all cases. Among the values recorded in the device, weight (kg), fat mass (kg), lean body mass (kg), muscle mass (kg), bone mass (kg), total body water (kg), BMI (kg/m^2^), and fat percentage (%) for whole-body analysis were extracted. Fat mass, lean body mass, muscle mass, bone mass, and total body water were divided by the square of height to calculate the fat mass index (FMI), LMI, muscle mass index (MMI), bone mass index (BNI), and total body water index (TWI), respectively. AC was measured at the umbilical level by wrapping a tape measure around the waist and recording the measurement in centimeters to one decimal place during exhalation.

### Blood examination for lipid-related indicators

Participants were not required to fast at the time of peripheral blood sample collection. Total cholesterol (TC), triglycerides (TG), and high-density lipoprotein cholesterol (HDLC) levels were measured in peripheral blood samples, from which the HDLC to TC (TC/HDLC) and HDLC to TG (TG/HDLC) ratios were calculated as lipid-related indicators.

### Wrist-worn device

The number of steps and activity and sleep durations were monitored using the Fitbit Ace 2 (Fitbit, California, United States) device. The participants were provided with the device to wear for 11 days to monitor their number of steps and activity and sleep durations. To obtain data from the wrist-worn device, an account linked to each participant’s research ID was created before wearing the device. After retrieving the devices, the data were synchronized with the Fitbit data server. Data files in JSON format were retrieved from each account. The C# language was used to aggregate the Fitbit record data and generate daily data, which were used for data aggregation at the 0 AM delimiter for steps and overall physical activity and sleep durations, recorded between 18:00 in the evening and 12:00 the next day. Physical activity intensity was categorized into four levels: light, moderate, vigorous, and sedentary. To ensure data reliability, we excluded data from the first and last 3 days from the analysis. Weekday and holiday data were retained.

**Fig. 1:**
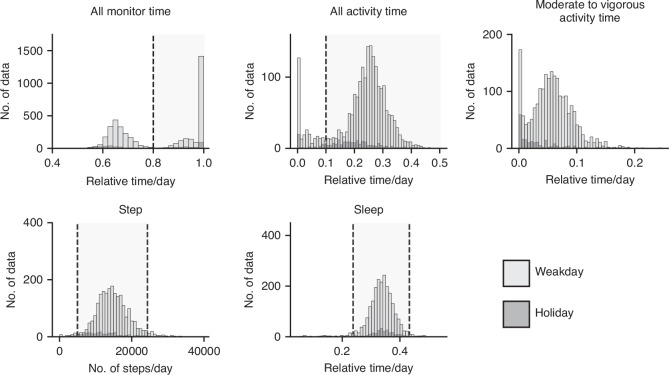
Screening activity and sleep duration data collected using wrist-worn devices. Bar graphs show the distribution of each measurement and highlight the extracted gray areas: values exceeding 0.8 for all monitor time, values exceeding 0.1 for all activity time, and values within the mean ± 2 standard deviations for daily step counts and sleep duration.

Daily activity and sleep duration data were rigorously screened to ensure data quality and completeness using the following criteria: for activity assessment, the sum of duration for all activity categories should be equal to the total monitored duration, ideally 1440 min per day. Thus, the measurement duration for each activity category was divided by 1440 min to calculate the “relative duration/day” proportion (range: 0–1). Two criteria were applied to ensure sufficient monitoring duration: first, the relative duration/day for the total monitored duration should be greater than 0.8; second, the relative duration/day for total activity duration (light, moderate, and vigorous) should be greater than 0.1. Daily data for the number of steps taken and sleep duration for each participant were obtained using the same software. Data points within ±2 standard deviations (SDs) of the mean were considered acceptable. The relative duration per day was calculated for sleep and activity durations. Only data meeting all specified criteria were included in the analysis, with the mean calculated for each participant for each year. The distribution of each measurement value and the screening results are presented in Fig. 1.

### Statistical analysis

#### Establishment of reference value models for BC indices (BCIs)

The following analyses were performed using Python (Python Software Foundation, https://www.python.org/) version 3.9 and R (R Foundation for Statistical Computing, https://cran.r-project.org/) version 4.1. The standard model of the BCI was established as follows: Three scores were calculated from the mean and SD of each BCI according to age (integer) and sex: the mean and mean ± 1 SD. For each score, a third-order polynomial regression model was assigned to age (integer) using the numpy.polyfit in Python. From this model, the mean and SD of age to the first decimal place were calculated, while five BCI datasets were standardized.

#### Standardization of anthropometric measurements

The *z*-scores for height, weight, and BMI were calculated using Excel-based Clinical Tools for Growth Evaluation of Children (taikakushisu_v3.3:　http://jspe.umin.jp//medical/files_chart/taikakushisu_v3.3..xlsx) published by the Japanese Society for Pediatric Endocrinology.

#### Dealing with missing data

The number of missing values for each measurement was 3 (0.33%) for AC, 110 (12.00%) for lipid-related indicators, 86 (9.38%) for the number of steps, and 134 (14.61%) for activity and 183 (19.96%) for sleep durations, and all were less than 20%. The missing values were addressed using three methods: (1) the *k*-nearest neighbors algorithm, implemented using KNNImputer (n_neighbors=5) in Python; (2) a multiple imputation approach, employing multivariate imputation by chained equations (MICE) using the mice package in R; and (3) complete case analysis after deleting missing values. The MICE was conducted under the following conditions: 20 input data sets, 50 imputations per data set, predictive mean matching as the imputation method, a fixed seed value of 1234, and inclusion of all variables with missing values in the imputation process. Among these, the *k*-nearest neighbors algorithm was used for the main analysis, while the MICE and complete case analyses were performed as sensitivity analyses to assess the robustness of the findings.

#### Cluster analysis and group comparison

Unsupervised hierarchical clustering was performed using seaborn.clustermap function (method = Ward) in Python, with standardized indices used to identify distinct subgroups. Additionally, *k*-means clustering was performed as a sensitivity analysis. For multiple group comparisons, the chi-square test, one-way analysis of variance (ANOVA), and calculation of the Pearson correlation coefficient were performed using scipy.stats in Python. In multiple regression analyses, cluster classification, measurement year, sex, and age were analyzed as explanatory variables, with sex considered a dummy variable as follows: boys, 0; and girls, 1. After handling missing data using the *k*-nearest neighbors method and complete case analysis, a mixed-effects model was constructed using the lme4 package in R, treating participant registration numbers as a random effect. In contrast, for the multiple imputation approach, a mixed-effects model could not be applied due to compatibility limitations with the mice package, and the registration numbers were therefore not included as a random effect. Statistical significance was set at *P* < 0.05.

## Results

### Characteristics of the study participants

**Fig. 2:**
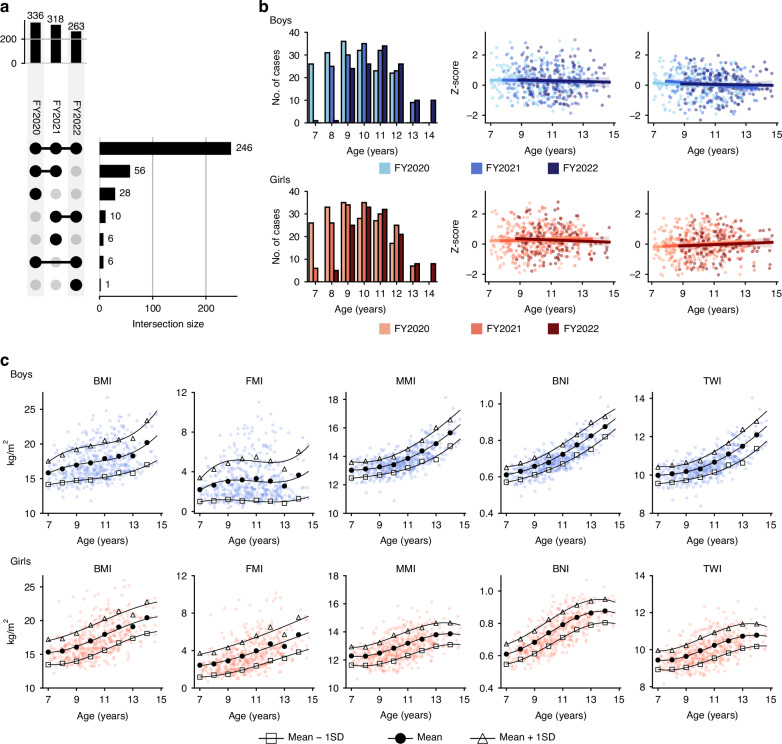
Overview of the participants and establishment of reference value models for body composition indices. **a** Upset plot indicating the matrix layout for all intersections across the three measurement years, sorted by size. Circles in the matrix represent sets included in the intersections. **b** Bar graphs indicating the number of participants in each measurement year, alongside a scatter plot displaying *z*-scores of height and weight. The regression line and the 95% confidence interval are shown in the scatter plot. **c** Scatter plots with polynomial approximation curves (third degree) are displayed. FY financial year, BMI body mass index, FMI fat mass index, MMI muscle mass index, BNI bone mass index, TWI total body water index, SD standard deviation.

Throughout the 3 years, 917 BC datasets were collected (336 in FY2020, 318 in FY2021, and 263 in FY2022; 455 boys and 462 girls). The age distribution based on sex for each year, and the number of participants in each of the 3 years and the number of times they underwent measurements are presented in Fig. 2a. The overall mean (SD) *z*-scores for height and weight of the participants who underwent BIA measurements were 0.29 (0.93) and 0.04 (0.86) for boys and 0.29 (0.90) and −0.02 (0.87) for girls, respectively, which were normative. There was no apparent change in their distribution by age over the 3-year period from 2020 to 2022 (Fig. 2b).

### Establishment of reference models for BCIs

Reference value models for BCIs were constructed using statistical procedures (Fig. 2c). Based on these models, we calculated the mean and SD of the BCIs for each age (to one decimal place) separately for boys and girls (Supplementary Table S1) and standardized all values, transforming each index into its respective *z*-score. The correlations between the *z*-score of BMI and the *z*-scores of BCIs were as follows: *r* = 0.96 with FMI, *r* = 0.77 with LMI, *r* = 0.77 with MMI, *r* = 0.71 with BNI, and *r* = 0.77 with TWI. This standardization process enabled the comparison of consistent and uniform indicators across sex and age groups and provides a normalized framework for further analysis and interpretation.

### Cluster analysis identified five subpopulations

**Fig. 3:**
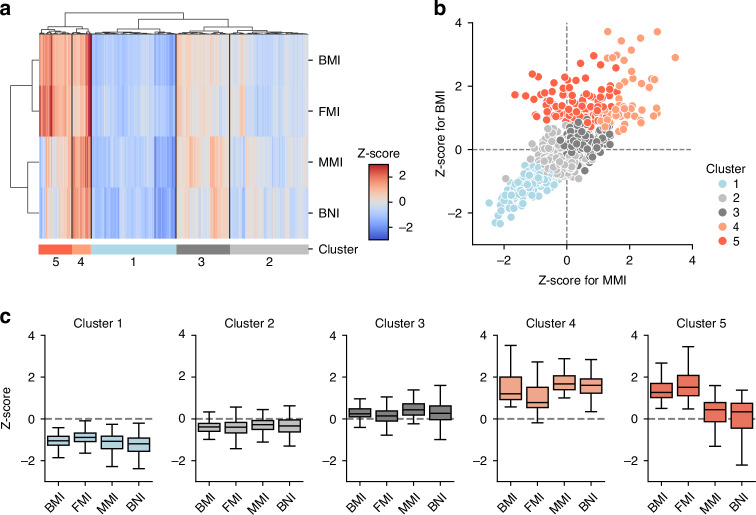
Clustering analysis using body mass index and body composition indices. **a** Heatmap showing the results of unsupervised hierarchical clustering analysis. The horizontal axis represents individual cases, and the vertical axis represents the body mass index and body composition indices. **b** Boxplots show the *z*-score distribution for body mass index and body composition indices in each cluster. **c** Scatter plot showing body mass index and muscle mass index. BMI body mass index, FMI fat mass index, MMI muscle mass index, BNI bone mass index.

**Fig. 4:**
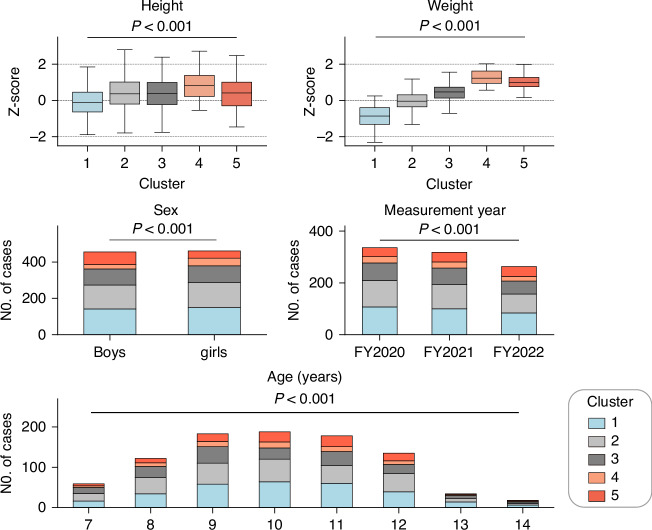
Comparison of characteristic data between the five clusters. *P* values for *z*-score of height and weight were determined using analysis of variance, and the chi-square test was applied to analyze sex, year of measurement, and age.

We attempted to profile participants using these standardized indices. First, we examined all possible combinations of the five BCIs, observing an almost perfect positive correlation among *z*-scores of LMI, MMI, and TWI (LMI and MMI: *r* = 0.9994; MMI and TWI: *r* = 0.9982; LMI and TWI: *r* = 0.9984). Subsequently, we selected MMI as a representative of this set. Next, unsupervised hierarchical clustering was performed using *z*-scores of BMI, FMI, MMI, and BNI and, the participants were classified into five clusters (Fig. 3a). The distribution of each index between the clusters is presented in Fig. 3b. The five clusters exhibited distinct characteristics: cluster 1 exhibited values below the mean for all indices; clusters 2 and 3 showed values close to the mean (with cluster 2 slightly below the normal range and cluster 3 slightly above it); cluster 4 had values above the mean for all indices; and cluster 5 was characterized by *z*-scores of BMI and FMI values above the mean, whereas *z*-scores of MMI and BNI hovered around the mean. When examining the relationship between *z*-scores of BMI and MMI (Fig. 3c), clusters 1–4 were found to be distributed linearly, whereas cluster 5 deviated from this distribution. Comparisons between clusters of other characteristics are shown in Fig. 4. The *z*-scores of height and weight for each cluster showed a trend similar to those of MMI and BNI; they increased from clusters 1 to 4 and slightly decreased in cluster 5. Significant differences in sex were observed among the five clusters and different proportions in clusters 4 and 5, with no noticeable differences in the year of measurement or age.

As a sensitivity analysis, five clusters were identified using the *k*-means method. The clustering patterns were mostly consistent with those obtained from hierarchical clustering (Fig. S1a, b), and similar distributions of BMI and BCIs were observed across clusters (Fig. S1c).

### Differences in biological and activity characteristics between clusters

**Fig. 5:**
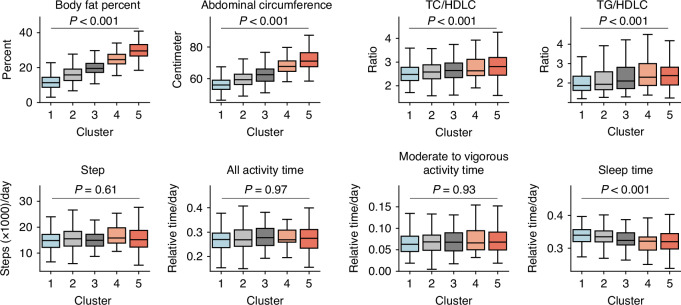
Differences among five clusters of health-related indicators and lifestyle data. Boxplots showing the distribution of biological characteristics in each cluster. *P* values were calculated by one-way analysis of variance. TC total cholesterol, TG triglycerides, HDLC high-density lipoprotein cholesterol.

**Fig. 6:**
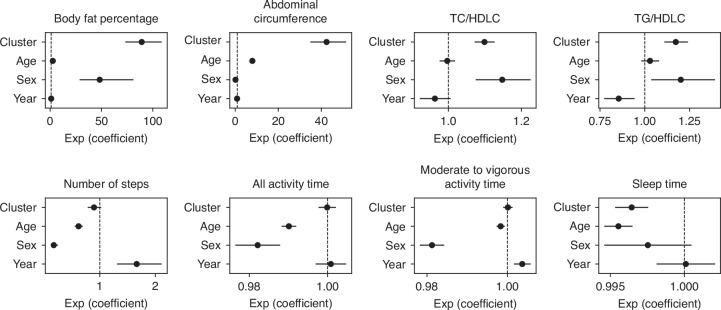
Identification of factors affecting health-related indicators and lifestyle data. Multiple regression analysis was performed for each parameter, with cluster, age, sex, and year of measurement as explanatory variables. The *x*-axis shows the regression coefficients exponentiated by natural logarithms. The black dot represents the exponentiated regression coefficient, and the horizontal bar indicates the 95% confidence interval. TC total cholesterol, TG triglycerides, HDLC high-density lipoprotein cholesterol.

To assess how these five clusters reflected other biological information, we evaluated AC, serum cholesterol level, activity and sleep durations, and number of steps. The mean and standard error of the data imputed via the *k*-nearest neighbors method for the missing values of these eight indicators are presented in Supplementary Table S2. Univariate ANOVA for the five clusters revealed statistically significant differences in body fat percent (BFP), abdominal circumference (AC), TC/HDLC ratio, TG/HDLC ratio, and sleep duration with missing values imputed using the *k*-nearest neighbors algorithm (Fig. 5). Notably, BFP and AC increased linearly from clusters 1 to 5. The TC/HDLC and TG/HDLC ratios were higher in clusters 4 and 5 than in clusters 1–3. Sleep duration was lower in clusters 4 and 5 than in clusters 1–3. However, no statistically significant differences were observed in the activity duration or number of steps per day. However, these eight indicators may be influenced by sex, age, and year of measurement; therefore, multiple regression analysis was conducted using these four factors, including cluster classification as explanatory factors (Fig. 6). Significant differences based on cluster classification for BFP, AC, TC/HDLC ratio, TG/HDLC ratio, and sleep duration were also observed in the multiple regression analysis. Similar results were obtained with a dataset addressing missing values using the MICE and complete case analysis (Fig. S2).

## Discussion

In this study, 917 BIA measurements were performed in 353 children throughout a 3-year period. In addition to BC measurements, anthropometry, AC, blood cholesterol levels, and activity and sleep durations were assessed. Reference models for BCIs were established and participants were grouped into five clusters based on clustering analysis. We identified differences in health-related indicators and lifestyles among the five clusters.

The present study provides BCI reference value models for the typical output variables of BC measurement: fat mass, lean mass, muscle mass, bone mass, and water mass. The reference values for BC and BCI in children have been reported in several studies using dual x-ray absorptiometry^5,20–26^ and BIA.^27–32^ The trends of the FMI and MMI were similar to those of the studies using the BIA method.^27,29,30^ Furthermore, although muscle is the largest component of body weight, all correlation analyses of BMI and BCI indicated a strong correlation between FMI and BMI, surpassing that of LMI or MMI. This association has also been observed in previously reported data.^27^ Thus, the consistency between our data and previous reports indicates the generality and usefulness of our established reference value model.

Our cluster analysis yielded several novel findings. Firstly, cluster 1, characterized by low BMI, FMI, MMI, and BNI, was the most common of the five clusters. Although cluster 1 cannot be defined as sarcopenia because there are no clear criteria for sarcopenia in children, a lower BMI was associated with lower overall BC components. Clusters 2 and 3 had approximately an average proportion; however, classifying them is meaningful, considering that cluster 2 is closer to cluster 1 while cluster 3 is closer to subpopulations with high BMI, i.e., cluster 4 or 5. Of the two subpopulations, cluster 4 had evenly high FMI, MMI, and BNI, whereas cluster 5 had high FMI but average MMI and BNI. Recently, Torres-Costoso et al. proposed five clusters using LMI and FMI.^18^ Among them, clusters of high FMI and average LMI existed, consistent with cluster 5 in our study. However, they further identified a population with average adiposity and high lean body mass, which was not included in our analysis, probably because of the different age groups (10–18 years) targeted by them. Overall, our clustering analysis revealed an association between BMI and BCIs and suggested that BC gradually changed and was heterogeneous at a high BMI.

Herein, we explored the relationship between cluster classification and biological characteristics. BFP, AC, TC/HDLC, and TG/HDLC as health-related indicators showed linear changes in clusters 1–5. TG/HDLC and TC/HDLC serve as predictive markers of insulin resistance^33,34^ and metabolic syndrome^35–37^ in childhood. These factors tended to increase as clusters changed from 1 to 5, suggesting that cluster 5 was the population with the highest health risk. Regarding lifestyle, sleep duration was shorter in clusters 4 and 5, while lipid markers were higher in 5 than in other clusters. Sleep deprivation is associated with various health problems.^38–41^ However, due to the cross-sectional nature of this study, the ability to infer causal relationships between BC, lifestyle factors, and health outcomes is limited. Longitudinal studies are needed to clarify these causal relationships. Additionally, while this study identified a group (cluster 5) considered to be potentially high risk, it did not directly examine specific strategies to mitigate these risks. Future studies should investigate the impact of these clusters on health and whether maintaining appropriate lifestyle habits contributes to improved health status. The comprehensive approach employed in this study for analyzing BC may serve as a valuable tool for identifying children at elevated risk of health problems and for guiding preventive interventions aimed at improving their living environments and overall health status.

This study has several limitations. First, the study was conducted during the COVID-19 pandemic, which may have influenced children’s physical activity and mental health. Reports have indicated that the pandemic and associated lockdown had a negative impact on children’s physical activity.^42–44^ According to the WHO guidelines,^45^ an average of 60 min of moderate-to-vigorous physical activity per day throughout the week is recommended for children. In the present study, only 83.4% of participants maintained the recommended activity level (81.8% in FY2020, 83.6% in FY2021, and 85.2% in FY2022), although this is only known within the observation period of less than 1 week. These results suggest that the validity of our findings was preserved, despite the impact of the pandemic. However, further studies conducted outside the context of the pandemic are necessary to confirm these findings. Second, the study participants were students attending a high-education-level national university-affiliated elementary school, which may have introduced bias stemming from specific educational and social backgrounds. Although the height and weight of the participants were the same as the average for Japanese children, the generalizability of the study findings may be limited. Additionally, we did not collect medical history or information on children’s health status. Therefore, we cannot definitively confirm that all children were in complete health. However, all children were attending regular school activities without any apparent medical conditions. Thus, it is reasonable to assume that the participants were generally healthy children. Accordingly, the data obtained from this population may be considered valid and useful as reference values for BC in children. Third, the use of wrist-worn devices for activity and sleep duration monitoring resulted in a high percentage of excluded data due to device removal during certain activities, potentially underestimating vigorous activity levels. For example, some participants removed the wrist-worn device during activities such as intense exercise or contact sports such as basketball or soccer. This means that some aspects of vigorous activity may not be adequately measured, which could explain why we found no significant differences between clusters in terms of activity parameters. Finally, most prior studies on TG/HDLC and TC/HDLC as predictive markers of insulin resistance and metabolic syndrome have collected blood samples in fasting conditions.^35–37^ However, in this study, blood samples for lipid-related indicators were collected without fasting, as fasting conditions are typically recommended for such measurements. Furthermore, detailed information on dietary intake or nutritional habits, which are closely related to metabolic status, was not evaluated. These factors may have affected the results and cannot be completely ruled out as potential sources of bias.

In conclusion, through our analysis, we categorized generally healthy children into five clusters based on their anthropometric data and BC components. BMI and BCIs were balanced in clusters 1–4, but imbalanced in cluster 5. Additionally, cluster classification was associated with health indicators and sleep duration, particularly in cluster 5, which had higher BFP, larger AC, higher lipid-related indices, and shorter sleep duration. Overall, our findings emphasize the importance of measuring BC using anthropometric measurements to assess children’s health status. Further research is required. to understand the underlying causes of the imbalances observed in cluster 5 and explore potential interventions aimed at reducing health risks in this population.

## Supplementary information

Supplemental material

Supplementary Table S1

Supplementary Table S2

## Data Availability

The data used in this study are unsuitable for public deposition due to ethical restrictions and the legal framework of Japan. Sharing of this data is prohibited by the Act on the Protection of Personal Information (Act No. 57 of 30 May 2003, amendment on 9 September 2015) to publicly deposit data containing personal information. Ethical Guidelines for Medical and Health Research Involving Human Subjects enforced by the Japan Ministry of Education, Culture, Sports, Science and Technology and the Ministry of Health, Labour and Welfare also restricts the open sharing of the epidemiologic data.
