# Supplementary material for: Cluster analysis with body composition data for health risk assessment in children
Source: Pediatr Res. 2025 Oct 30;99(5):1764–71. doi: 10.1038/s41390-025-04447-6 (PMC13221299; doi:10.1038/s41390-025-04447-6)
Supplement: Supplementary file 1 — Supplemental material [file 41390_2025_4447_MOESM1_ESM.pdf]

Cluster analysis with body composition data for health risk assessment in children

Authors:
Wataru Kudo; Keita Terui; Midori Yamamoto; Rieko Takatani; Aya Hisada; Chisato Mori; Tomoro Hishiki; Kenichi Sakurai

This document file includes:

Fig. S1. Sensitivity analyses of cluster analysis.

Fig. S2. Sensitivity analyses of multiple regression analysis.

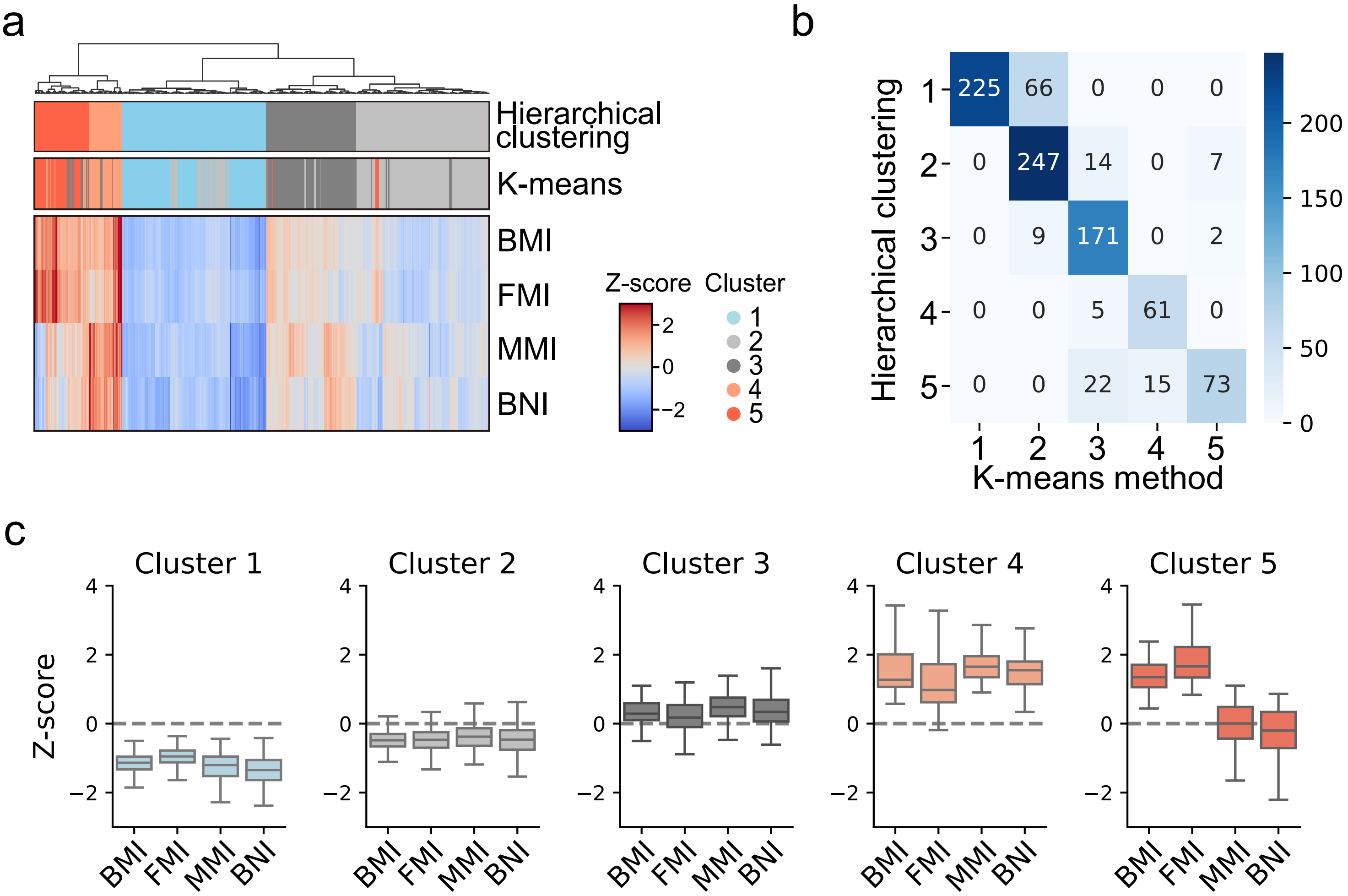

Fig. S1. Sensitivity analyses of cluster analysis.

(a) Heatmap shows the standardized values of body composition variables, with samples grouped by hierarchical clustering with Ward method (top dendrogram). Colored bars above the heatmap indicate cluster assignments by both hierarchical clustering (top row) and k-means (second row). (b) Heatmap shows the degree of correspondence between hierarchical clustering and k-means method. The numbers in the cells at the intersection of the two axes indicate the number of samples classified into the corresponding clusters by both clustering methods. The color bar indicates the number of samples. (c) Boxplots show the z-score distribution for body mass index and body composition indices in each cluster by k-means methods.

BMI, body mass index; FMI, fat mass index; MMI, muscle mass index; BNI, bone mass index.

a. Multivariate imputation by chained equations

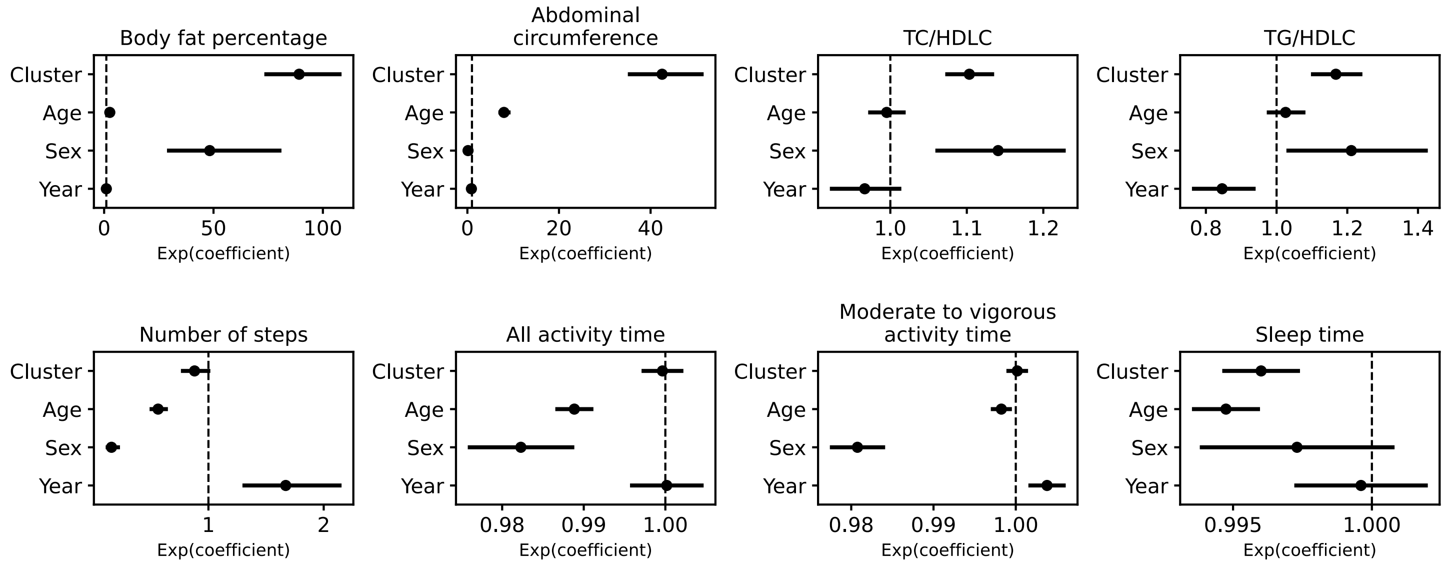

b. Complete case analysis

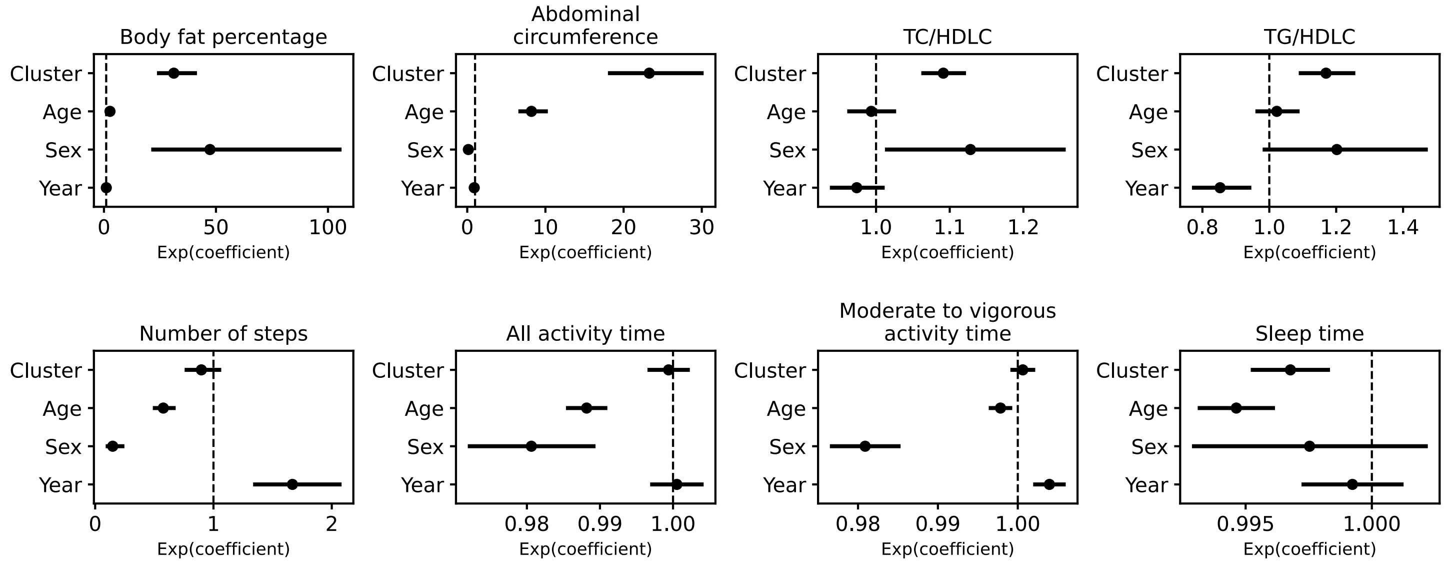

Fig. S2. Sensitivity analyses of multiple regression analysis.

Forest plots show the results of multiple regression analyses for each parameter, with cluster age, sex, and year of measurement as explanatory variables. Missing values were addressed using the MICE method (a) and complete case analysis (b).

TC, total cholesterol; TG, triglycerides; HDLC, high-density lipoprotein cholesterol.
